# Examining transcriptional changes to DNA replication and repair factors over uveal melanoma subtypes

**DOI:** 10.1186/s12885-018-4705-y

**Published:** 2018-08-14

**Authors:** Melanie Kucherlapati

**Affiliations:** 1 Department of Genetics, Harvard Medical School, Boston, 02115 MA USA; 2 Department of Medicine, Division of Genetics, Brigham and Women’s Hospital, 77 Avenue Louis Pasteur NRB 160B, Boston, 02115 MA USA

**Keywords:** Replication, Repair, Expression, Uveal melanoma

## Abstract

**Background:**

Uncontrolled replication is a process common to all cancers facilitated by the summation of changes accumulated as tumors progress. The aim of this study was to examine small groups of genes with known biology in replication and repair at the transcriptional and genomic levels, correlating alterations with survival in uveal melanoma tumor progression. Selected components of Pre-Replication, Pre-Initiation, and Replisome Complexes, DNA Damage Response and Mismatch Repair have been observed.

**Methods:**

Two groups have been generated for selected genes above and below the average alteration level and compared for expression and survival across The Cancer Genome Atlas uveal melanoma subtypes. Significant differences in expression between subtypes monosomic or disomic for chromosome 3 have been identified by Fisher’s exact test. Kaplan Meier survival distribution based on disease specific survival has been compared by Log-rank test.

**Results:**

Genes with significant alteration include MCM2, MCM4, MCM5, CDC45, MCM10, CIZ1, PCNA, FEN1, LIG1, POLD1, POLE, HUS1, CHECK1, ATRIP, MLH3, and MSH6. Exon 4 skipping in CIZ1 previously identified as a cancer variant, and reportedly used as an early serum biomarker in lung cancer was found. Mismatch Repair protein MLH3 was found to have splicing variations with deletions to both Exon 5 and Exon 7 simultaneously. PCNA, FEN1, and LIG1 had increased relative expression levels not due to mutation or to copy number variation.

**Conclusion:**

The current study proposes changes in relative and differential expression to replication and repair genes that support the concept their products are causally involved in uveal melanoma. Specific avenues for early biomarker identification and therapeutic approach are suggested.

## Background

Comparatively uncontrolled replication carried out by highly evolutionarily conserved multiprotein complexes, is a process shared by all cancers. Although many other processes including immortality, epithelial to mesenchyme transition, telomere metabolism, metastasis etc. contribute to tumorigenesis, the summation of genomic alterations in a tumor must facilitate replication. Duplication in the transformed cell is achieved at the expense of decreased fidelity, making replication a focal point where heterogeneity is created that upon clonal selection leads to tumor expansion and survival. Several individual critical replication genes have been examined in The Cancer Genome Atlas (TCGA) efforts and auxiliary studies, with computational methods placing alterations into known pathways to identify potential targets for precision medicine. However, the behavior of replication factors as a group did not receive analogous systematic investigation, likely because they are thought of as being part of a process rather than a pathway.

TCGA has recently conducted an integrative analysis of uveal melanoma (UVM), and the data providing its molecular foundation are now available to the public as part of the “Rare Tumor Project” [1]. While having a low incidence of 5.1 per million [2] it is the most common intraocular malignancy. UVM is highly lethal, with 50% of patients developing metastatic disease followed by 6–12 month survival from metastatic diagnosis [3]. Datasets for whole exome sequencing (WES), whole genome sequencing (WGS), mRNA miRNA and lncRNA expression, DNA methylation, identification of immune infiltration, and detailed pathology with clinical outcome were generated. Using these data, the current study examines the status of small groups of genes with known biology in replication and repair at the genomic and transcriptional levels in UVM. Selected components from the Pre-replication, Pre-initiation, Replisome, DNA Damage Repair (DDR), and Mismatch Repair (MMR) complexes have been investigated for genomic and transcriptional changes across UVM clusters classified by TCGA.

Alterations indicative of replication stress (RS) correlating with aneuploidy, increased malignancy, and decreased survival have been observed, as well as changes to common replication components involved in bypass of replication stress. Generally RS is thought to be an early and strong driving force in tumorigenesis, and is seen over a broad spectrum of cancer types. It is defined as impediment to the replication fork that causes slowing or stalling of the replication machinery and is brought on by “exogenous” and “endogenous” factors [4]. Examples of exogenous causes are radiation, therapeutic treatment, and diet. Endogenous causes include nucleotide pool availability, DNA structures, protein-DNA complexes, reactive oxidation species, transcription and replication complex collision, mutation and expression alteration in tumor suppressor and oncogenes.

This study specifically identifies changes in expression across UVM subtypes to MCM2, MCM4, MCM5, CDC45, MCM10, CIZ1, PCNA, FEN1, LIG1, POLD1, POLE, HUS1, CHECK1, ATRIP, MLH3, and MSH6. Resulting implications for bio-marker and therapeutics are discussed, and a rationale given for the observed alterations.

## Methods

### Cluster and mutational analysis

The cluster analysis relied upon in this study was made by TCGA and is based on Somatic Copy Number Alteration (SCNA) [1]. Clusters are referred to as 1–4 throughout, they correlate with groups A-D by Royer-Bertrand et al. [5]. Mutational findings are based upon data generated by the TCGA Research network and can be found in cBioPortal [6–8].

### Differential expression analysis

In this report expression analysis is made by two algorithms, using the terms “differential” and “relative” to distinguish between them. Differential expression for UVM is defined as mRNA Z Scores (RNA-Seq by Expectation Maximization (RSEM)) (log2) compared to the expression distribution of each gene tumors that are diploid for the gene. The use of the diploid fraction is due to the lack of a normal control for RNA-Seq analysis (matched blood samples for each UVM case were available for DNA analysis). These data are calculated and made available by cBioPortal [8].

### Relative expression analysis

This study defines “relative” expression as mRNA Z Scores (RNA-Seq Reads per Kilobase of Transcript per Million Mapped Reads (RPKM)) (log2) comparison to the UVM tumor cohort average in its entirety, also known as the “average alteration level”. These data have been determined specifically for this study and were made for both mRNA and individual exons.

RNA-Seq derived exon expression levels were visualized in heat maps. The Gene Annotation File (GAF) “TCGA.hg19.June2011.gaf” [9] was used to create an exonStartStop.txt file for each gene tested which in turn was used to parse the “UVM.rnaseqv2__illuminahiseq_rnaseqv2__unc_edu__Level_3__exon_quantification__data.data.txt” file [14] to create an “exonRPKM.txt” file used for standard Z score generation. Both files, exonStartStop.txt and exonsRPKM.txt, were run through a verification step to confirm that the appropriate gene, TCGA barcodes, and RNA-Seq data were selected prior to their use. Exon start-stop sites from the exonStartStop.txt file were examined in Integrative Genome Viewer (IGV) [10, 11] using RNA-Seq data (9) from the same case to confirm the authenticity of the exon. The “Sashimi Plot” function in IGV was used to identify alternative splicing and isoforms from RNA-Seq data, with “minimum junction coverage” routinely set at “4”. Using “R” [12] Z scores were calculated for each exon of each gene by mean-centering with the average alteration level the log2 transformed RPKM values and dividing by the standard deviation, visualizing high (red), no change/no expression (white), and low (blue) and arranging data by UVM cluster assignments (1–4) in heat maps.

### Placement of cases into “high” and “low” expression groups

An output file containing Z scores for each exon was created and used to calculate an average Z score for each gene. This was regarded reasonable as structural variations to genes were found only with low frequency. UVM cases were sorted into two groups, those with average Z scores “above” and those “below” zero. After group designation, the cluster [1–4] each case belonged to was identified and the numbers of cases “high” and “low” for each cluster counted. Significant differences between “1 and 2” versus “3 and 4” were determined by Fisher’s exact (two tailed), using GraphPad Quick Calcs. Because very few genes and few cases (total *n* = 80, in each cluster *n* = 15 through 23) were examined, “q” values were not calculated, with the rationale that doing so might increase “type II” errors. Since placement of cases into “high” and “low” grouping was made relative to the total tumor cohort average, conjecturally, all cases “below” the tumor cohort average could also be “above” an average made from appropriate adjacent normal tissues, which was not available for RNA analysis in UVM samples. The procedure was evaluated using BAP1 and RPS19 as test genes.

### Clinical data and survival analysis

The TCGA UVM cohort was made up of eighty matched tumor and normal blood specimens. Tumors were obtained from patients that did not have previous systemic chemotherapy or focal radiation, with appropriate consent obtained from institutional review boards. A panel of five histopathologists with expertise in ocular pathology and melanoma, examined hematoxylin and eosin stained sections from paraffin embedded tumors defining tumor extent, cell morphology, pigmentation, mitotic index, and the presence of tumor-infiltrating lymphocytes and macrophages. The following information was also curated: “tumor status” (date of last contact), “vital status” (dead/alive), “date of last contact”, “date of death”, “cause of death”, “other cause of death”, “new tumor event after initial treatment”, “histology of new tumor event”, “site of new tumor”, “other site/new event”, “date of new event”, “additional surgery”, “additional treatment/radiation”, “additional treatment/pharmaceutical”.

In principal four types of survival analysis can be made with TCGA clinical data. “overall survival” defined as the period from date of diagnosis until death from any cause, “progression-free interval” from date of diagnosis until the occurrence of an event in which the patient with or without the tumor does not get worse, “disease-free interval” date of diagnosis until first recurrence, and “disease specific survival” diagnosis date until death from the specific cancer type. All UVM survival curves constructed for this study were “disease specific survival” curves, as recommended by TCGA Pan Cancer Guidelines [13]. Kaplan Meier survival plots were constructed using GraphPad Prism 6.0 software. The Log-rank (Mantel-Cox) and Hazard Ratio tests were used to determine significance.

## Results

### BAP1 and RPS19

**Fig. 1:**
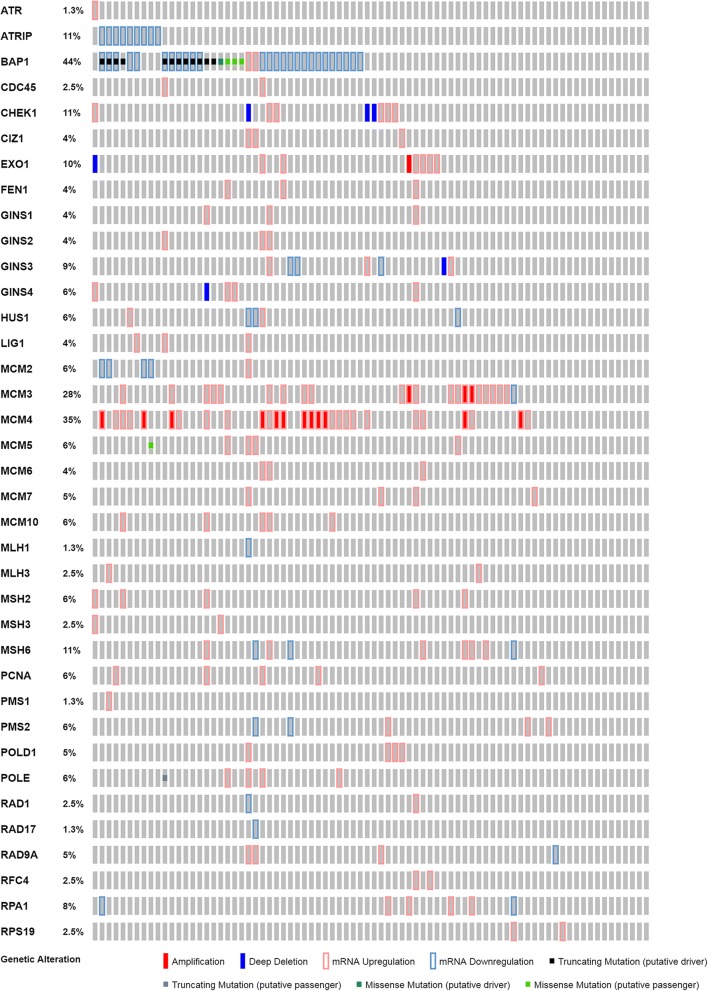
Oncoprint depicting mutation and expression alteration for selected genes involved in replication and repair in eighty UVM cases

A total of thirty-seven genes (Table 1) were observed for mutations and differential expression (Fig. 1). BAP1 and RPS19 differential and relative expression were compared (Table 2). Examination of BAP1 differential expression Z scores calculated by cBioPortal, showed a two-fold greater inclusion of cases “below” average expression. For this tumor type due to the relationship of monosomy 3 to subtypes and survival, the approach using an estimated reference (the diploid fraction) alters comparison to relative expression for genes located on chromosome 3. SCNA subtypes 3 and 4 are both monosomic for chromosome 3 and constitute approximately half the total tumor cohort. In contrast using RPS19, a gene which codes for a 40S Ribosome complex protein with cytological location at chromosome 19q13.2, showed no significant difference in relative expression found between the study method and cBioPortal values. UVM do not have significant SCNA for chromosome 19. The four additional cases found in “below” of cluster 3 are due to the use of RSEM verses RPKM. These results show incongruity between differential expression about an estimated normal value and relative expression about a tumor cohort average, when high numbers of cases are not diploid. It should be noted explicitly that presentation of the discrepancy is not meant to claim one set of calculations superior to the other, but to explain why additional calculations were made for relative expression.

**Table 1:** A selected list of genes associated with replication and DNA Repair

Gene Symbol	Description	Location
ATR	ATR Serine/Threonine Kinase	3q23
ATRIP	ATR Interacting Protein	3p21.31
BAP1	BRCA1 Associated Protein 1	3p21.1
CDC45	Cell Division Cycle 45	22q11.21
CHEK1	Checkpoint Kinase 1	11q24.2
CIZ1	CDKN1A Interacting Zinc Finger Protein 1	9q34.11
EXO1	Exonuclease 1	1q43
FEN1	Flap Structure-Specific Endonuclease 1	11q12.2
GINS1	GINS Complex Subunit 1	20p11.21
GINS2	GINS Complex Subunit 2	16q24.1
GINS3	GINS Complex Subunit 3	16q21
GINS4	GINS Complex Subunit 4	8p11.21
HUS1	HUS1 Checkpoint Clamp Component	7p12.3
LIG1	DNA Ligase 1	19q13.33
MCM2	Minichromosome Maintenance Complex Component 2	3q21.3
MCM3	Minichromosome Maintenance Complex Component 3	6p12.2
MCM4	Minichromosome Maintenance Complex Component 4	8q11.21
MCM5	Minichromosome Maintenance Complex Component 5	22q12.3
MCM6	Minichromosome Maintenance Complex Component 6	2q21.3
MCM7	Minichromosome Maintenance Complex Component 7	7q22.1
MCM10	Replication Initiation Factor	10p13
MLH1	MutL Homolog 1	3p22.2
MLH3	MutL Homolog 3	14q24.3
MSH2	MutS Homolog 2	2p21 -p16.3
MSH3	MutS Homolog 3	5q14.1
MSH6	MutS Homolog 6	2p16.3
PCNA	Proliferating Cell Nuclear Antigen	20p12.3
PMS1	PMS1 Homolog 1, Mismatch Repair System Component	2q32.2
PMS2	PMS1 Homolog 2, Mismatch Repair System Component	7p22.1
POLD1	DNA Polymerase Delta 1, Catalytic Subunit	19q13.33
POLE	DNA Polymerase Epsilon, Catalytic Subunit	12q24.33
RAD1	RAD1 Checkpoint DNA Exonuclease	5p13.2
RAD17	RAD17 Checkpoint Clamp Loader Component	5q13.2
RAD9A	RAD9 Checkpoint Clamp Component A	11q13.2
RFC4	Replication Factor C Subunit 4	3q27.3
RPA1	Replication Protein A1	17p13.3
RPS19	Ribosomal Protein S19	19q13.2

**Table 2:** Z Score Comparison Above and Below Zero, Differential versus Relative Expression

Gene ID	Differential Expression (cBioPortal)	Relative Expressionn (Study)	X^2^
	Cluster 1 Above/Below Average (*n* = 15)	Cluster 1 Above/Below Average (*n* = 15)	
BAP1	5/10	14/1	0.0007
RPS19	15/0	15/0	Identical
	Cluster 2 (*n* = 23)	Cluster 2 (*n* = 23)	
BAP1	13/10	22/1	0.0019
RPS19	10/13	10/13	Identical
	Cluster 3 (*n* = 22)	Cluster 3 (*n* = 22)	
BAP1	0/22	4/18	0.0359
RPS19	7/15	11/11	Not Different
	Cluster 4 (*n* = 20)	Cluster 4 (*n* = 20)	
BAP1	0/20	6/14	0.0079
RPS19	2/18	2/18	Identical

### Pre-replication and pre-initiation complexes

**Fig. 2:**
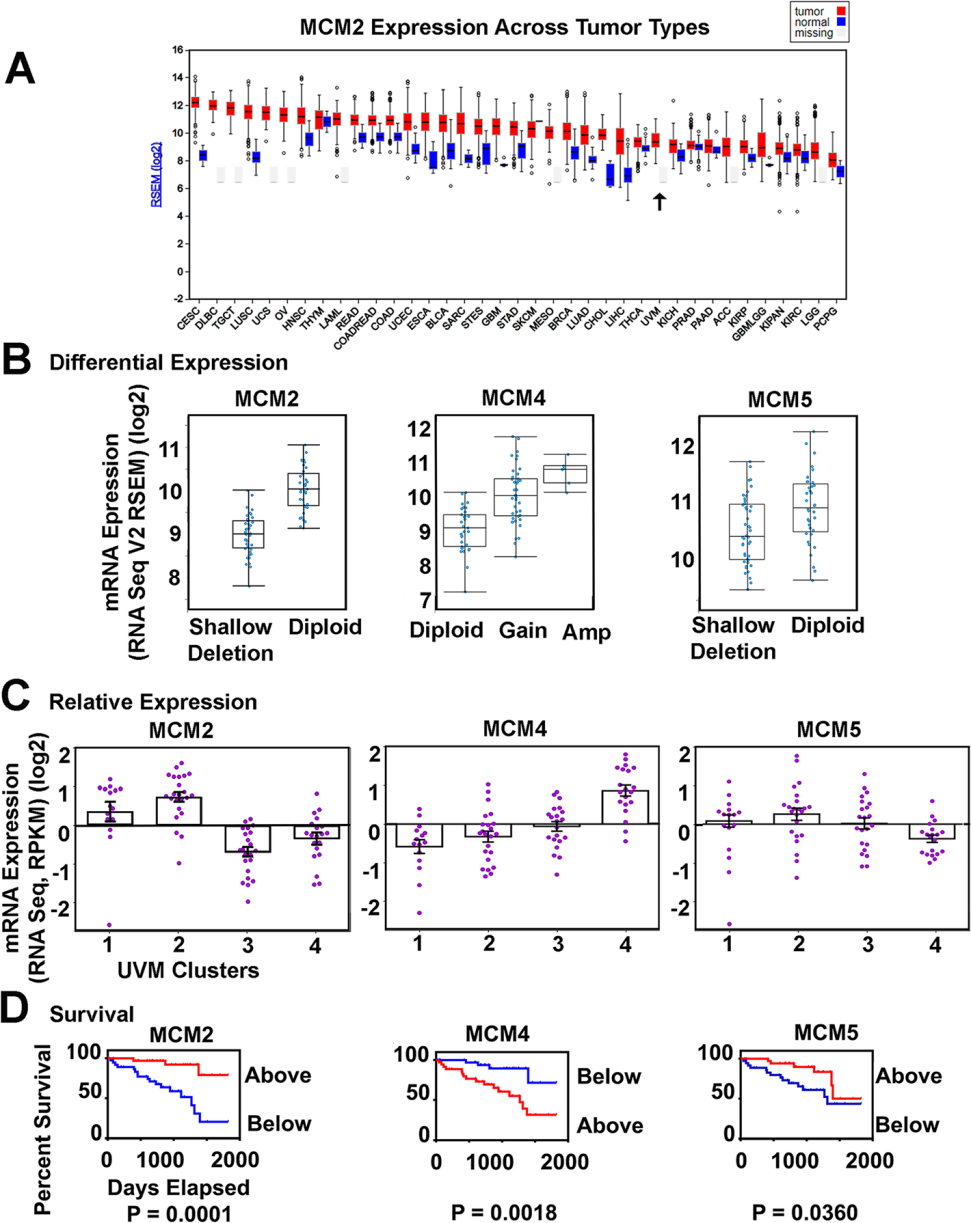
Alterations to the Mini Chromosome Maintenance (MCM) Helicase Complex **a** RNA- Seq abundance of MCM2 across tumor types, highest to lowest expression UVM (black arrow). **b** Differential Expression, mRNA expression z-scores (RNA-Seq V2 RSEM) (log2) for MCM2, MCM4, and MCM5, median and SD. Putative copy-number alterations are from GISTIC. **c** Relative Expression, mRNA Expression z-scores (RNA-Seq RPKM) (log2) organized across UVM clusters 1–4, mean and SEM. **d** Kaplan Meier Survival Plot, cases “above” (red) “below” average (blue) zero z-score, Log-rank test

**Fig. 3:**
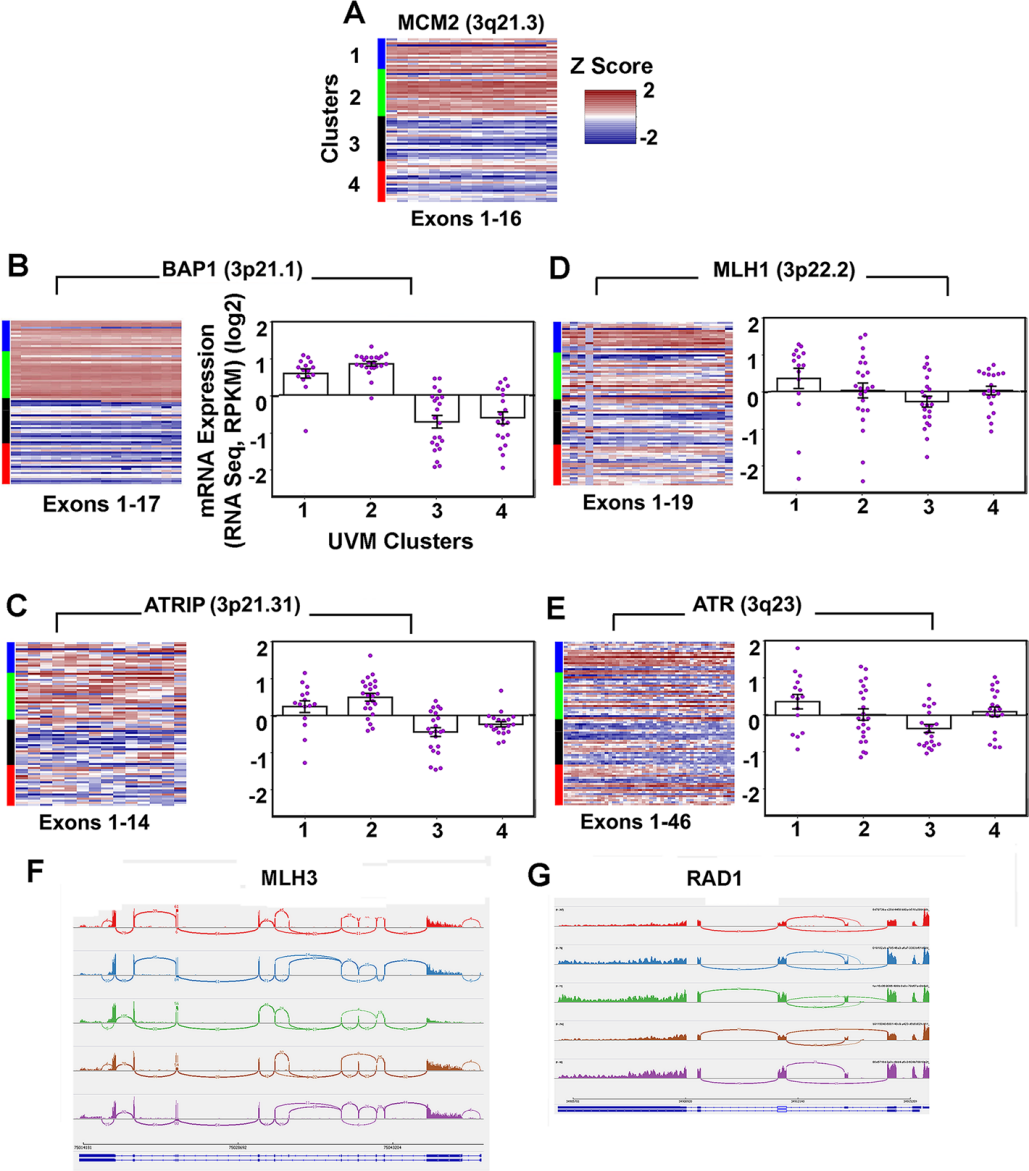
mRNA Expression from Chromosome 3 Genes. **a** Heatmap of MCM2 mRNA relative expression for exons 1–16, organized by UVM subtype; high expression (red), no change (white), low (blue). **b** Heatmap of BAP1 exons 1–17 with corresponding relative expression scatter plot (mean, SEM). **c** ATRIP (**d**) MLH1 (**e**) ATR. **f** Sashimi plot (from Integrative Genome Viewer) depicting alternative splicing of MLH3. **g** Sashimi plot, RAD1

Differential expression of all Mini-Chromosome Maintenance (MCM) helicase components of the pre-replication complex is increased over a wide range of tumor types. The results for MCM2 (3q21.3) specifically are given in Fig. 2 [14]. Relative expression profiles document MCM2 drops below the tumor average significantly in UVM clusters 3 and 4 (*P* = 0.0001) (Table 3), correlating with increased malignancy and decreased disease specific survival (*P* = 0.0001). Half of the helicase complex components (MCM2–7) are located on chromosomes found by TCGA to have copy number alterations that include monosomy chromosome 3, 8q and 6p gains. A comparison of other genes in this study found on chromosome 3 indicates expression levels do not always simply correlate with SCNA (Fig. 3), reflecting the TCGA finding that expression subtypes are only partially concordant with SCNA subtypes [1].

**Table 3:** Relative Expression and Survival Correlation of Pre-Replication and Pre-Initiation Complex Factors

Gene	UVM Cluster 1 Above/Below Average (%/%)	UVM Cluster 2 Above/Below Average (%/%)	UVM Cluster 3 Above/Below Average (%/%)	UVM Cluster 4 Above/Below Average (%/%)	*P* Value 1&2 vs 3&4 Fisher’s Exact Two Tailed	Total Cases Above Average	Total Cases Below Average	Kaplan Meier Worse Survival	Kaplan Meier Log-rank
	*n* = 15	*n* = 23	*n* = 22	*n* = 20					
Pre-Replication Complex									
MCM2	12/3 (80/20)	20/3 (87/13)	3/19 (14/86)	5/15 (25/75)	< 0.0001	40	40	Below	0.0001
MCM3	7/8 (47/53)	10/13 (43/57)	7/15 (32/68)	12/8 (60/40)	1.0000	36	44	NA	0.5514
MCM4	2/13 (13/87)	6/17 (26/74)	11/11 (50/50)	18/2 (90/10)	< 0.0001	37	43	Above	0.0018
MCM5	9/6 (60/40)	16/7 (70/30)	11/11 (50/50)	4/16 (20/80)	0.0133	40	40	Below	0.0360
MCM6	6/9 (40/60)	9/14 (39/61)	15/7 (68/32)	19/1 (95/5)	0.0002	49	31	NA	0.1809
MCM7	3/12 (20/80)	17/6 (74/26)	11/11 (50/50)	11/9 (55/45)	1.0000	42	38	NA	0.2614
Pre-Initiation Complex									
CDC45	5/10 (33/67)	6/17 (26/74)	14/8 (64/36)	15/5 (75/25)	0.0007	40	40	NA	0.4409
GINS1	5/10 (33/67)	8/15 (35/65)	6/16 (27/73)	15/5 (75/25)	0.1074	34	46	NA	0.1774
GINS2	8/7 (53/47)	11/12 (48/52)	14/8 64/36)	9/11 (45/55)	0.8229	42	38	NA	0.1965
GINS3	11/4 (73/27)	11/12 (48/52)	8/14 (36/64)	11/9 (55/45)	0.2735	41	39	NA	0.6131
GINS4	6/9 (40/60)	12/11 (52/48)	11/11 (50/50)	9/11 (45/55)	1.0000	38	42	NA	0.5714
MCM10	3/12 (20/80)	9/14 (39/61)	9/13 (41/59)	15/5 (75/25)	0.0261	36	44	NA	0.2409
CIZ1	12/3 (80/20)	15/8 (65/35)	13/9 (59/41)	2/18 (10/90)	0.0019	42	38	Below	0.0032

Conversely, MCM4 (8q11.21) expression is increased in clusters 3 and 4, with escalation correlating to worse survival that correlate with SCNA gains to chromosome 8q in cluster 4 (*P* = 0.0018) (Fig. 2, Table 3). MCM5 (22q12.3) has lower relative expression in UVM clusters 3 and 4, with cases having worse survival. For MCM5, the *P* values are significant but less convincing (*P* = 0.036).

**Fig. 4:**
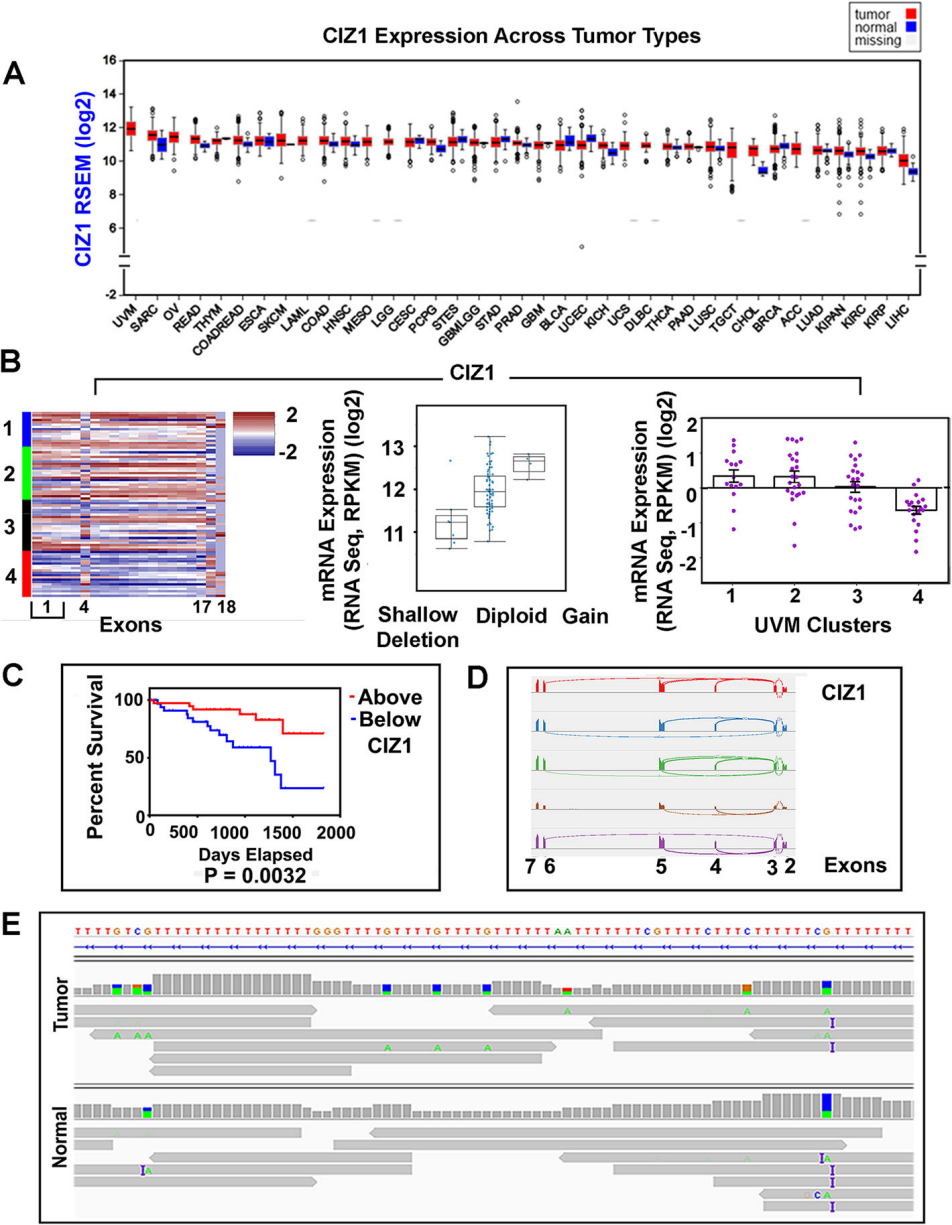
CIZ1 (**a**) Differential Expression, across tumor types. **b** Heatmap of CIZ1 relative expression with exon1 alternative splicing and variation to exons 4, 17, and 18. CIZ1 Differential Expression and CIZ1 relative expression. **c** Kaplan Meier survival plot (Log-rank test). **d** Sashimi plot, exon 4 skipping. **e** CIZ1 Intron 3 mononucleotide repeat (hg19:130,950,210-130,950,372), adenine insertions (purple bar)

Other genes associated with the pre-replication complex and examined in this study include CDC45, GINS1–4, MCM10, and CIZ1. CDC45 and MCM10 were both highly expressed in the higher risk subtypes, but did not correlate with survival changes (Table 3). GINS1–4 relative expression was not altered across the clusters. CIZ1 [15–20], had highly significant difference between the clusters, with lower relative expression correlating to higher risk subtypes and decreased survival (*P* = 0.0019) (Fig. 4 b-c, Table 3). Evidence of exon 4 skipping previously seen in Ewing tumor [21] and Lung Cancer [22] and in the C terminal region was found in the CIZ1 heatmap (Fig. 4b) and in RNA-Seq using the Sashimi Plot function in IGV (Fig. 4d). CIZ1 intron 3 contains a mononucleotide repeat previously associated with exon 4 skipping mechanistically, and hypothesized to be the result of MMR deficiency [21]. Fifty out of 80 UVM tumors had low pass WGS for tumor and normal counterpart tissues as well as tumor RNA-Seq. These cases were examined in IGV for alterations to Intron 3 (hg19:130,950,210-130,950,372). Almost all tumors and normal samples had some alteration (Fig. 4e). Clear identification of Microsatellite Instability (MSI) from sequencing artefact wasn’t possible. These results are discussed more fully below.

### Replisome, DNA damage response, mismatch repair proteins

**Fig. 5:**
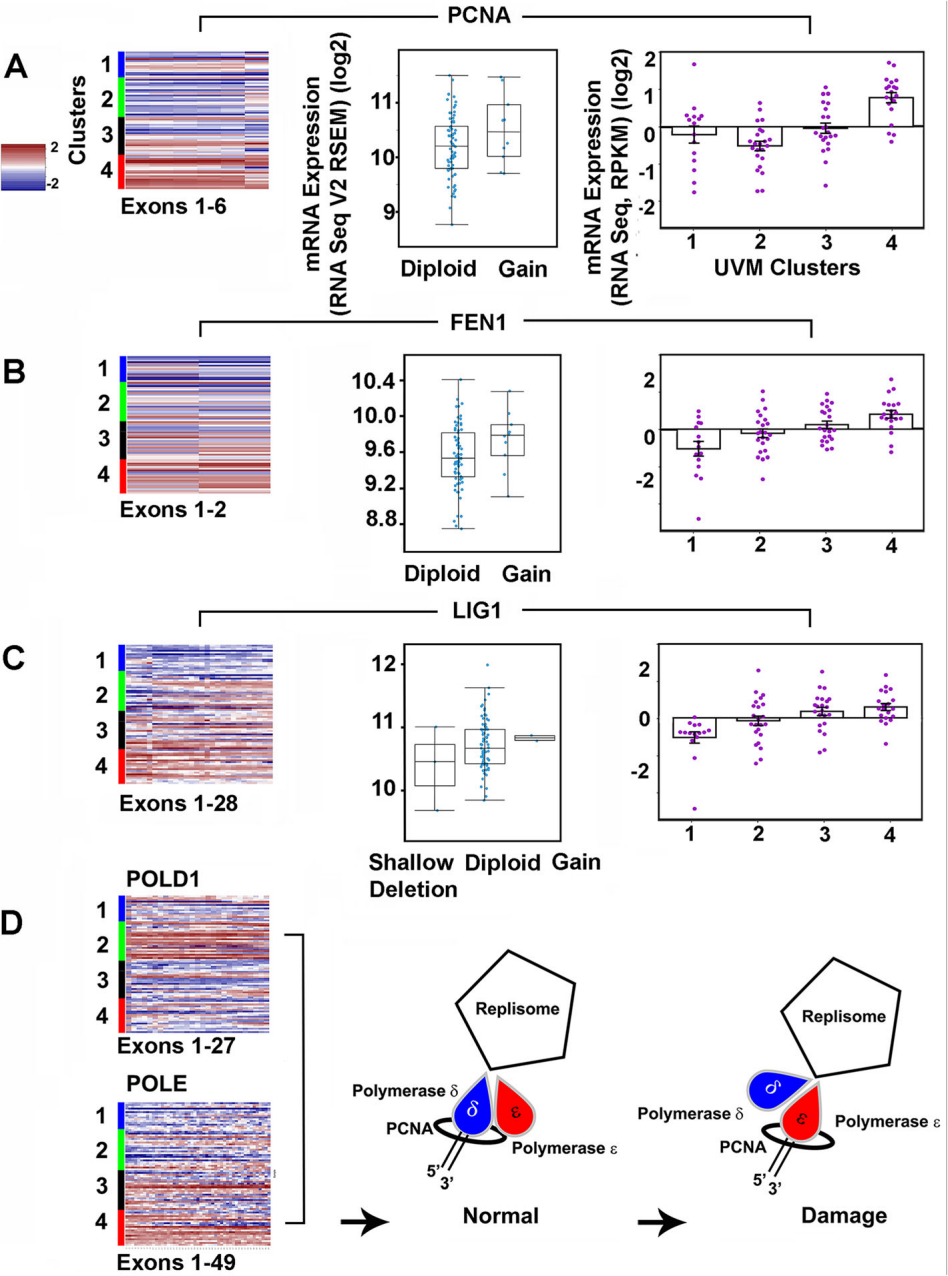
Replisome Components **a** Heatmap depicting Proliferating Nuclear Cell Antigen (PCNA) relative expression across mRNA; Differential expression (median, SD); Relative Expression (mean, SE). **b** Flap endonuclease (FEN1). **c** Ligase 1 (LIG1). **d** POLD1 and POLE relative expression with schematic

PCNA, FEN1, LIG1, and HUS1 were found to have increased relative expression across clusters 1–4 (Fig. 5). PCNA and HUS1 increase correlated significantly with worse survival, FEN1 and LIG1 did not (Table 4). RFC4 and RPA1did not have differences (data not given).

**Table 4:** Relative Expression and Survival Correlation of Replisome and DNA Damage Response Factors

Gene	UVM Cluster 1 Above/Below Average (%/%)	UVM Cluster 2 Above/Below Average (%/%)	UVM Cluster 3 Above/Below Average (%/%)	UVM Cluster 4 Above/Below Average (%/%)	*P* Value 1&2 vs 3&4 Fisher’s Exact Two Tailed	Total Cases Above Average	Total Cases Below Average	Kaplan Meier Worse Survival	Kaplan Meier Log-rank
	*n* = 15	*n* = 23	*n* = 22	*n* = 20					
Replisome									
PCNA	8/7 (40/60)	4/19 (17/83)	8/14 (36/64)	17/3 (85/15)	0.0147	37	43	Above	0.0280
FEN1	5/10 (33/67)	9/14 (39/61)	11/11 (50/50)	17/3 (85/15)	0.0132	42	38	Above	0.0884
LIG1	1/14 (7/93)	10/13 (44/56)	16/6 (73/27)	15/5 (75/25)	0.0001	42	38	NA	0.2590
POLD1	7/8 (47/53)	17/6 (74/26)	7/15 (32/68)	6/14 (30/70)	0.0067	37	43	Below	0.0597
POLE	4/11 (27/73)	14/9 (61/39)	11/11 (50/50)	16/4 (80/20)	0.1761	45	35	Above	0.0990
DNA Damage Response									
HUS1	7/8 (47/53)	9/14 (39/61)	12/10 (55/45)	18/2 (90/10)	0.0124	46	34	Above	0.0278
RAD9A	9/6 (60/40)	10/13 (43/57)	13/9 (59/41)	16/4 (80/20)	0.1105	48	32	NA	0.8086
RAD1	8/7 (53/47)	10/13 (43/57)	10/12 (45/55)	16/4 (80/20)	0.2609	44	36	NA	0.1076
RAD17	8/7 (53/47)	9/14 (39/61)	9/13 (41/59)	16/4 (80/20)	0.2624	42	38	NA	0.5653
ATR	11/4 (73/27)	10/13 (44/56)	6/16 (27/73)	13/7 (65/35)	0.5021	40	40	NA	0.8707
CHEK1	4/11 (27/73)	8/15 (35/65)	12/10 (55/45)	15/5 (75/25)	0.0041	39	41	NA	0.3072
ATRIP	11/4 (73/27)	19/4 (83/17)	6/16 (27/73)	2/18 (10/90)	0.0001	38	42	Below	0.0001

POLD1 relative expression was significantly different in clusters 1 and 2 compared to 3 and 4, survival correlation was not (*P* = 0.06, Log rank). POLE expression was not significantly different across clusters 1 through 4. Examining POLD1 and POLE clusters 3 & 4 to each other however, showed significant difference in the number of cases “above” and “below” the mean (*P* = 0.0042), indicating clusters 3 and 4 had more cases with higher than average POLE expression than POLD1 (Fig. 5).

RAD1, RAD9A, and RAD17, members of the DDR complex did not have significant expression or survival differences. RAD1 showed evidence of alternative splicing involving exon 3 previously reported as a natural isoform (Fig. 3). ATRIP was significantly decreased in the more malignant clusters 3 and 4 and this decrease correlated with survival decrease (Table 4), HUS1 was increased.

Mismatch repair proteins MLH3 and MSH6 had significant expression differences across clusters but no survival advantages or disadvantages (Table 5). MLH1 relative expression did not completely behave as anticipated from simple correlation with chromosome 3 loss (Fig. 3). RNA-Seq data was examined for several MMR genes in IGV and further evidence of exon skipping and alternative splicing was found for MLH3. An isoform was identified by Sashimi plots that had deletions in both exons 7 and exon 5 simultaneously (Fig. 3f).

**Table 5:** Relative Expression and Survival Correlation of Mismatch Repair Genes

Gene	UVM Cluster 1 Above/Below Average (%/%)	UVM Cluster 2 Above/Below Average (%/%)	UVM Cluster 3 Above/Below Average (%/%)	UVM Cluster 4 Above/Below Average (%/%)	*P* Value 1&2 vs 3&4 Fisher’s Exact Two Tailed	Total Cases Above Average	Total Cases Below Average	Kaplan Meier Worse Survival	Kaplan Meier Log-rank
	*n* = 15	*n* = 23	*n* = 22	*n* = 20					
MLH1	11/4 (73/27)	13/10 (57/43)	7/15 (32/68)	11/9 (55/45)	0.0781	42	38	NA	0.9990
MLH3	6/9 (40/60)	9/14 (39/61)	12/10 (55/45)	16/4 (80/20)	0.0242	43	37	NA	0.3170
MSH2	7/8 (47/53)	12/11 (52/48)	9/13 (41/59)	16/4 (80/20)	0.5004	44	36	NA	0.7404
MSH3	8/7 (53/47)	11/12 (48/52)	9/13 (41/59)	16/4 (80/20)	0.5004	44	36	NA	0.3593
MSH6	4/11 (27/73)	13/10 (57/43)	10/12 (46/54)	16/4 (80/20)	0.0357	43	37	NA	0.7278
PMS1	6/9 (40/60)	9/14 (39/61)	9/13 (41/59)	16/4 (80/20)	0.1165	40	40	NA	0.3997
PMS2	8/7 (53/47)	15/8 (65/35)	11/11 (50/50)	14/6 (70/30)	1.0000	48	32	NA	0.7110
EXO1	8/7 (53/47)	10/13 (43/57)	4/18 (18/82)	13/7 (65/35)	0.6526	35	45	NA	0.4349

## Discussion

**Fig. 6:**
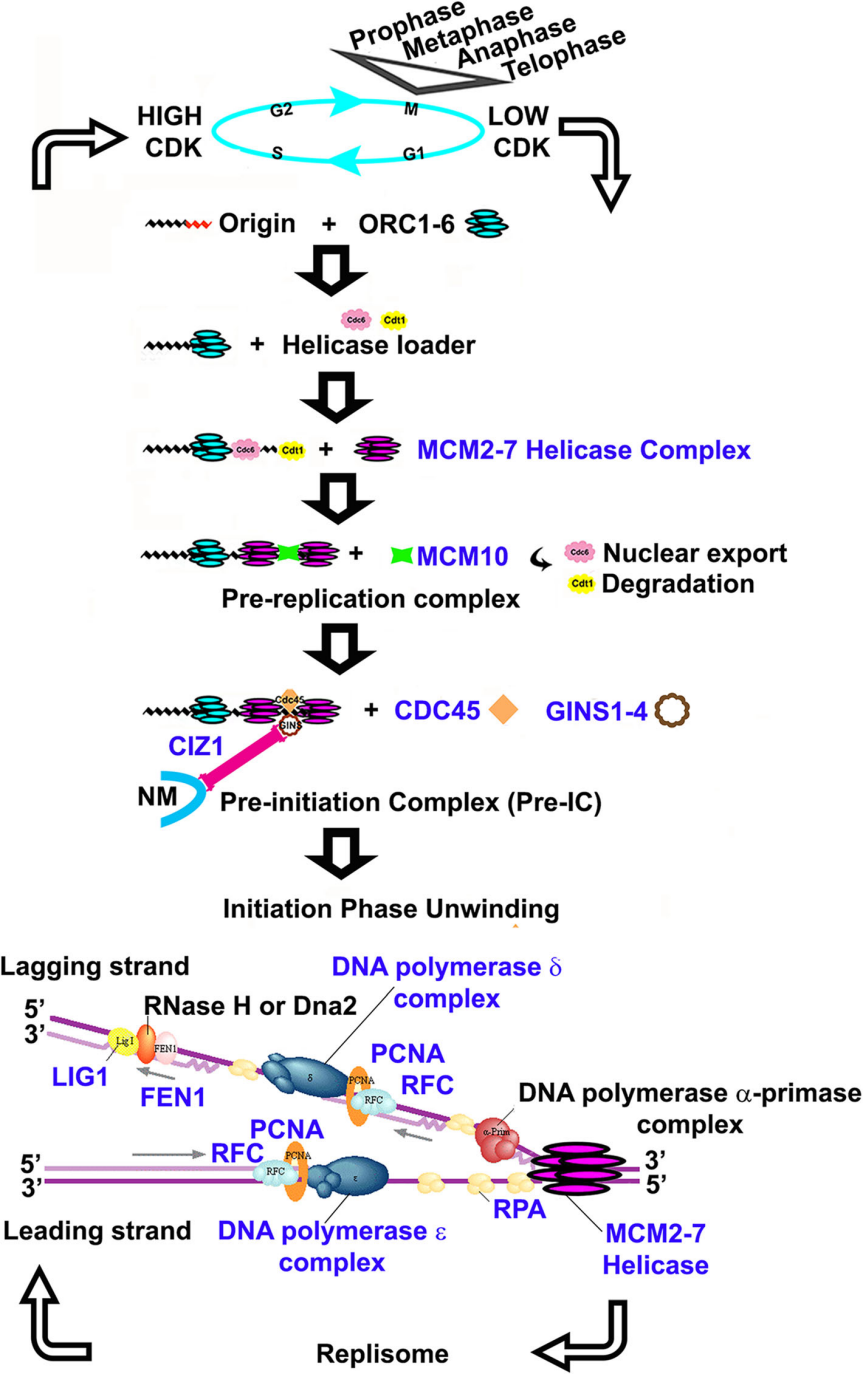
Schematic Representation Depicting Selected genes in the context of Replication. Selected genes in study (*blue*) [54]

**Fig. 7:**
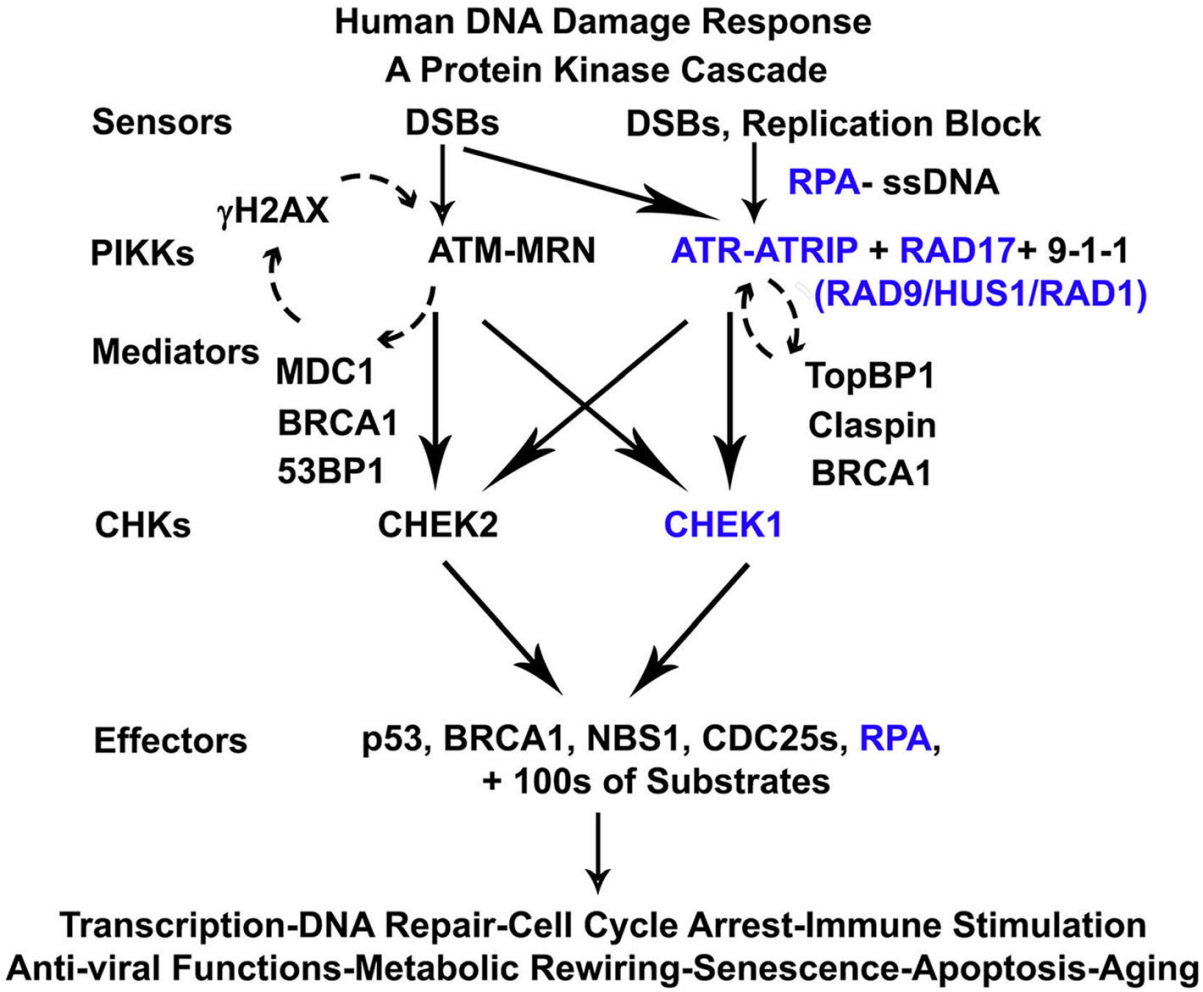
DNA Damage Response (DDR) Pathway. Selected genes in study (*blue*) [55]

**Fig. 8:**
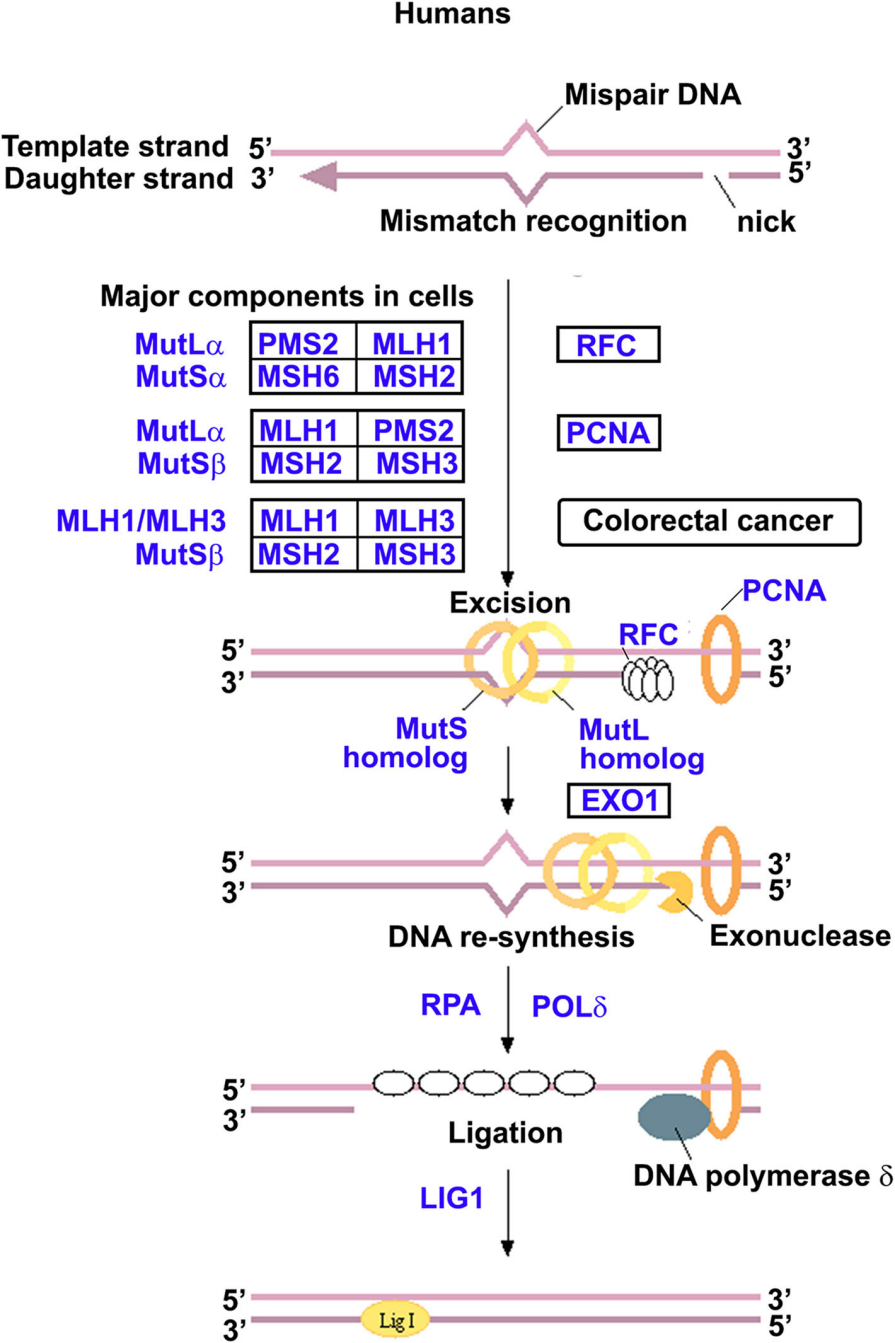
Mismatch Repair (MMR) Pathway. Selected genes in study (*blue*) [54]

Clinical treatments for UVM fall under the broad categories of radiation, surgery, laser, and novel therapies for primary disease [3]. Despite measures, metastatic tumor cells persist in up to half of patients leading to death. It is probable that until therapy can halt the procession of the replication complex in a non-harmful tumor specific manner, that there will always be the potential for the selection of persistent cells. The rationale for focusing on replication and repair genes is to identify changes that directly account for the persistent tumor cells that implicitly make appropriate targets. The replisome is inherently highly processional and is kept under control by complexes of proteins that determine where, when, and how it functions in normal development, homeostasis, and in the tumor phenotype. This study suggests the replisome is increasingly unfettered by the inactivation of controlling complexes such as the origin of replication proteins, DDR, and MMR complexes as UVM progresses in malignancy. Schematic diagrams are presented (Figs. 6, 7 and 8) identifying selected genes of those complexes examined, and how they are thought to function normally.

Expression alteration to genes that create RS was found in untreated UVM, clearly indicating therapeutic radiation was not responsible for its initiation. On the basis of high frequency, the earliest mutations in UVM are thought to be to the guanine nucleotide binding proteins (GTPases) GNAQ and GNA11. It is a plausible hypothesis that alteration to these genes would directly or indirectly result in alterations to nucleotide pool availability in the nucleus, providing an early and endogenous cause of RS. Thought to be an early and driving event in cancer generally, there has been suggestion RS might be therapeutically targetable [23, 24].

This study specifically found changes to the MCM helicase complex. MCM2–7 components effectually act to “license” the firing of origins of replication during G1-phase of the cell cycle, determining the multiple nascent origins that replicate during S phase. A recent comprehensive review has been made by Riera et al. [25]. The complex is thought to load in excess, with dormant origins becoming active when nearby replication forks have stalled functionally serving as backup. Mouse models deficient in MCM components have high rates of cancer and genome instability [26–30], which supports the concept of relative deficiency of MCM factors in the more malignant UVM subtypes. Decrease in MCM2 is thought to reduce replication initiation in gene rich early replicating regions, and to increase genetic damage at a subset of gene rich locations [31] without changing the replication rate. Another putative function for MCM2 due to its histone binding and chaperone capacity may be to orchestrate histone dynamics during replication [32], and it has also been linked conceptually to transcription [33]. UVM appear to have MCM concentration alterations including decreased MCM2, increased MCM4 and decreased MCM5 that correlate with increased malignancy and decreased survival. Overexpression of MCM4 has been found previously in multiple transformed cell lines and numerous tumor types [34, 35]. Multiple kinase signaling pathways are known to target MCM4. It is thought to mediate the repression of late origin firing and provide an intrinsic regulatory domain for signal integration coordinating origin activation and replication fork progression under RS [36]. The unbalanced expression of MCM2–7 individual components likely affects the quantitative amount of functional helicase available, lowering the ability to load in excess and contributing to unregulated replication.

Once the pre-replication complex is augmented with proteins CDC45 and GINS1–4 it is designated as a “pre-initiation complex”, CIZ1 (also known as Cip1-interacting zinc finger protein 1) is thought to function in this context [20]. This interesting gene plays a role in cell cycle control and replication by interaction with Cip1/Waf1 [15], and coordinating cyclin E and A functions in the nucleus [17]. It is required for cells to enter into S phase [20]. CIZ1 physically tethers to non-chromatin nuclear structures in the nuclear matrix, and co-localizes in immune-histochemical studies with PCNA [15]. Two functionally distinct domains exist, an N-terminal domain involved in replication and a C-terminal domain comprising the nuclear matrix anchor [16]. Targeted depletion of transcripts decreases proliferation in vitro [15]. Differential expression plots show the gene is most highly expressed in UVM (Fig. 4). Relative expression across the TCGA SCNA subtypes shows clusters 1, 2, and 3 have CIZ1 expression above the alteration level and cases below are primarily in subtype 4. CIZ1 must be available in enough quantity in subtype 4 to provide the replication function. Relative paucity and alternative splicing likely creates less than optimum conditions for nuclear anchor functioning. Almost all UVM cases examined show evidence of exon 4 alteration previously reported in other cancers as well as alteration to the carboxyl end of the protein. Variant mRNA created by alternative splicing are found in cancers and other disorders that appear to alter protein localization [19]. Variant CIZ1 has recently been published as a circulating biomarker for early-stage lung cancer [22] suggesting the same might be possible for UVM patients.

The findings for CIZ1 led to identification a mononucleotide repeat in Intron 3 with possible MSI. Almost no evidence points to the involvement of MMR components as drivers for UVM, and examination of mutation rate does not show logarithmic elevation typical of MMR deficient tumors. Exon skipping was observed in MLH3 (MLH3 ∆ exon5/∆ exon7), a protein that interacts with MLH1 directly. MLH3 ∆ exon7 has been previously described [37] and in that study the isoform lacked MLH1 binding activity suggesting a mechanism for partial MMR defect in UVM. In addition to playing a role in MMR, MLH3 also has a meiotic phenotype. Evidence of MSI was observed in both tumor tissue and its normal counterpart. Mononucleotide tracks are difficult to sequence and the frequency of MSI found is implausibly high, however, some examples genuinely appear have alterations in the germline that also appear in the tumor. Interestingly, the differential expression plot across tumor types also shows CIZ1 expression is not much different between tumors and their normal tissues quantitatively (Fig. 4), unlike MCM2 for example (Fig. 2) supporting the concept of possible predisposition.

Reviewing relative expression for PCNA (Proliferating nuclear cell antigen), FEN1 (Flap endonuclease 1), and LIG1 (Ligase 1) an increase was found that implied their involvement in overcoming RS. These components of the replisome lacked mutation and SCNA but correlated none-the less with increased expression suggesting transcriptional mechanisms were used to overcome RS and fork collapse in UVM progression. PCNA is a ring shaped homo-trimer that encircles DNA [38]. It interacts competitively with many other factors at the PIP motif [39, 40] and is an essential co-factor for DNA polymerases during replication. It is involved in repair processes and can also be modified post-translationally by phosphorylation, ubiquitination, SUMO proteins, ISGylation, Acetylation, and S-nitrosylation. Each modification has corresponding biological functions that include proliferation, MMR inhibition, translesion synthesis, homologous recombination, genomic stability, and apoptosis, respectively. One of its major roles is to promote tolerance to DNA damage during replication [41]. Because of its role in proliferation, PCNA is a target for cancer therapy. Several small molecules that either block PCNA-Polδ or PCNA trimer formation have been identified with proliferation-inhibitory effects in vitro [38, 42–44].

Two polymerases, POLD1 and POLE, important in the replication of B form DNA were selected for observation. The relative decrease in POLD1 expression in subtypes 3 and 4 and increase in POLE support a recently proposed model [45] in which a switch to POL ɛ and away from POL δ occurs upon DNA damage. The behavior of POL α and specialized polymerases recently hypothesized to assist the replication machinery in the prevention of replication stress [4] has not yet been examined.

Replication stress activates DDR which prevents DNA damage from becoming fixed during replication and passed on in mitosis [46]. It does this in part by coordinating cell cycle control and providing a pause for repair, and in some cases by triggering apoptosis. Dysregulated DDR in a tumor cell contributes to progressive genomic instability that is cancer enabling, and can make the tumor dependent on alternative repair pathways such as base excision repair (BER) that may be targetable (e.g. Poly ADP ribose Polymerase (PARP) inhibitors). In this study we find evidence of dysregulated DDR in the more malignant subtypes. Examining individual components of DDR revealed a significant decrease in relative expression of ATRIP (ATR Interacting Protein) in clusters 3 and 4 [47–51]. PARADIGM and MARINa algorithms previously used to examine the pathway, found protein abundance for DDR components increased suggesting DDR was active in monosomy 3/BAP1 impaired UVM [1]. ATRIP is located at 3p21.31, very close to BAP1 at 3p21.1. Interestingly as mentioned in the TCGA study [1], recent reports suggest that BAP1 itself may function in DDR [52, 53].

## Conclusions

In summary, this study examines selected replication and repair factors in UVM with known biology for expression differences with and without mutation across TCGA SCNA subtypes. Differences in expression have been observed that support the concept that these genes and their products are causally involved in UVM. These data suggest further avenues of research for biomarker identification and therapeutic approach.

## Abbreviations

BER: Base Excision Repair
DDR: DNA Damage Repair
GAF: GO Annotation File
IGV: Integrative Genomics Viewer
MCM: Mini-Chromosome Maintenance
MMR: Mismatch Repair
MSI: Microsatellite Instability
PARP: Poly ADP ribose Polymerase
RPKM: Reads per Kilobase of transcript per million mapped reads
RS: Replication Stress
RSEM: RNAseq by Expectation Maximization
SCNA: Somatic copy number alterations
TCGA: The Cancer Genome Atlas
UVM: Uveal Melanoma
WES: Whole Exome Sequencing
WGS: Whole Genome Sequencing
